# Predictive Factors for Second-Line Therapy in Metastatic Renal Cell Carcinoma: A Retrospective Analysis

**DOI:** 10.15586/jkcvhl.2017.59

**Published:** 2017-03-21

**Authors:** Hendrik Eggers, Philipp Ivanyi, Mareike Hornig, Viktor Grünwald

**Affiliations:** Department of Hematology, Hemostasis, Oncology, and Stem Cell Transplantation, Hannover Medical School, Hannover, Germany

**Keywords:** clinical decision making, predictive markers, renal cell cancer, second-line therapy, targeted therapy

## Abstract

Currently, about 50% of patients with metastatic renal cell carcinoma (mRCC) receive a second-line therapy. Therefore, the choice at each subsequent treatment line remains an important issue. In this retrospective study, we sought to identify pretreatment clinical parameters that could predict the likelihood of a patient receiving a second-line therapy. One hundred and sixty-one mRCC patients who received targeted therapy were evaluated. Descriptive statistics, Kaplan–Meier overall survival (OS), Cox regression, and binary logistic regression models were used for data analysis. Second-line therapy was given to 105 patients (65%). Patients with grade 1 tumor received second-line therapy more frequently than those with grade 2/3 tumors (P = 0.03). Only tumor grade was significantly different between patients receiving, or not receiving, second-line treatment. Median OS was significantly superior in patients receiving second-line therapy (32 versus 14 months; P = 0.007; hazard ratio [HR], 1.75; P = 0.008), patients with grade 1 tumors (130 versus 29 months in G2/3 tumors; HR, 3.85; P = 0.009), and in patients without early tumor progression (41 versus 11 months; HR, 5.04; 95% confidence interval [CI], 3.06–8.31; *P* < 0.001). In binary logistic regression, we identified early progression to be significantly associated with a higher probability of not receiving a second-line therapy (HR, 2.50; 95% CI, 1.01–6.21; P = 0.048). This study hypothesizes that pretreatment grade and early progression are predictive parameters for the selection of patients for second-line therapy.

## Introduction

Targeted therapies have become the mainstay of treatment for metastatic renal cell carcinoma (mRCC), currently achieving a median overall survival (OS) of approximately 30 months (1). The recent improvements in targeted therapies enable clinicians to offer patients several lines of agents. However, in contrast to the expanded therapeutic armamentarium, only 42%–57% of mRCC patients receive a second-line therapy (2, 3). During the cytokine era of mRCC therapy, the Memorial Sloan Kettering Cancer Center (MSKCC) score proved to be a reliable predictor of patients receiving first-line immunotherapy with poor, intermediate, and good prognosis (4). Heng et al. (5) validated a modified prognostic scoring system, based on the MSKCC, for the era of first-line targeted therapies. The International Metastatic Renal Cell Carcinoma Database Consortium (IMDC), primarily designed for risk-assessment prior to initiating first-line therapy, recently validated it for risk prediction in second-line therapy. The IMDC score is also based on the MSKCC score and includes the following variables to stratify mRCC patients into poor, intermediate, and good outcome: Karnofsky Performance Status, platelet count, neutrophil count, hemoglobin concentration, serum calcium concentration, and time from diagnosis to treatment (6).

Apart from established risk stratification scores, there are various hematological, genetic, and anatomical parameters that have been analyzed for their predictive impact. For example, although metastatic tumor burden in three- dimensional volumetric analysis does not predict survival of mRCC patients, a composite biomarker score consisting of five biomarkers in the blood was significantly associated with progression-free survival (PFS) in mRCC patients treated with everolimus (7–9). The systemic inflammation response index based on widely available laboratory findings (hemoglobin concentration and lymphocyte-to- monocyte ratio) predicts OS and seems to be a better option (10). On the other hand, a genetically based approach would be to analyze polymorphisms of vascular endothelial growth factor A (VEGF A) receptor for their association with PFS (11).

Although these parameters might be useful in predicting patient survival and treatment outcome, they cannot be used to make recommendations for specific therapeutic approaches. Moreover, there is no established model or single parameter prior to first-line therapy that could predict whether, or not, a patient would require a second-line targeted therapy. The aim of this study was to find pretreatment clinical parameters as a predictor of patients who will be capable to receive a second-line therapy. Such parameters might enable clinicians to provide patients with an optimized therapeutic approach.

## Materials and Methods

### Patients and treatment

Patients with mRCC, treated with targeted agents in our department (Department of Hematology, Hannover Medical School, Hannover, Germany), were identified retrospectively from medical records. All patients receiving tyrosine kinase inhibitors (TKI) as first-line targeted agents from 2005 to 2012 were eligible. There were no exclusion criteria. We defined first-line therapy as the first administered TKI within the treatment history for mRCC. Any other systemic therapy after first-line TKI treatment was defined as second-line therapy. Discontinuation of first-line treatment was due to progressive disease, therapy-limiting toxicity, or patient request. Second-line treatment was administered after discontinuation of first-line therapy irrespective of the reason for discontinuation. Patients were treated with second-line therapy when eligible according to applicable treatment guidelines and local standards that included ECOG status, laboratory findings, and patient approval. Early progression was defined as progressive disease within 6 months of start of first-line TKI therapy. Progression was defined by radiological evidence of disease progression, clinical signs of progression, or death from disease. Dose modifications were made according to the summary of medical product characteristics. Treatment and therapeutic monitoring, based on computed tomographic scans every 3 months, were applied according to guidelines and local standard. Clinical data were extracted from medical charts and collected in a database. Data were assessed by physicians and data managers. The database was last updated in April 2013. Patients’ data were assessed in an anonymized manner in concordance with recommendations of the local ethics committee and the declaration of Helsinki in its latest revised version.

### Statistical analyses

All patients were divided into two subgroups for comparison: patients who received second-line therapy and patients who did not. The data were analyzed and compared by either Mann–Whitney test for parameters with more than two variables or Fisher’s exact test for categorical data. OS was calculated by Kaplan–Meier analysis, and subgroups were compared by log-rank test. Uni- and multivariate Cox regression models were conducted to analyze the association between survival and administration of second-line therapy for different clinical and patient covariates. OS was defined from the time of first-line TKI treatment initiation until death, or last follow-up. Patients lost to follow-up were censored at time of last documented follow-up. Additionally, uni- and multivariate binary logistic regression analyses were performed to determine the usefulness of pretreatment clinical characteristics as predictors of ability to receive a second-line therapy. A hazard ratio (HR) describes the relative risk of not being able to obtain a second-line therapy. SPSS 21.0 was used for statistical analyses. A two-sided *P* < 0.05 was considered as statistically significant.

## Results

### Clinical characteristics

Within the observation period, we identified 161 mRCC patients treated with first-line TKI. The median follow-up was 33 (interquartile range, 11–40.5) months. At last follow-up, 115 patients were alive while 46 patients had died. One hundred and nine patients (67.7%) were men. The predominant subtype was clear cell carcinoma (134 patients; 83.2%; Table 1). Sunitinib was the most frequently administered first-line agent (77%), followed by sorafenib (14.9%), axitinib (5.0%), and pazopanib (3.1%). The performance status defined by ECOG was 0 in the majority of patients (68.3%) and 1 in 8.7% of patients. Prior immunotherapy was documented in 38.5% of patients. Although 55.9% of patients had no available record of an MSKCC risk score, most patients at onset of mRCC presented an intermediate risk score (n = 50; 31.1%; Table 1).

**Table 1.** Baseline parameters of all patients, and comparison of patients receiving second-line therapy with patients not receiving second-line therapy

	All	Second line	No second line	*P*-value
Patients	161	105	56	
Gender, n (%)				
Male	109 (67.7)	74 (70.5)	35 (62.5)	0.376
Female	52 (32.3)	31 (29.5)	21 (37.5)	
Histology, n (%)				
Clear cell	134 (83.2)	85 (81.0)	49 (83.2)	0.304
Papillary	7 (4.3)	6 (5.7)	1 (1.8)	
Others	7 (4.4)	6 (5.7)	1 (1.8)	
NE	13 (8.1)	8 (7.6)	5 (8.9)	
Grading, n (%)				
G1	9 (5.6)	9 (8.6)	0 (0)	*0.030*
G2/3	131 (81.3)	85 (80.9)	46 (83.1)	
NE	21 (13.0)	11 (10.5)	10 (17.9)	
T (2002), n (%)				
1	26 (16.1)	16 (15.2)	10 (17.9)	0.216
2	26 (16.1)	22 (21.0)	4 (7.1)	
3	68 (42.2)	42 (40.0)	26 (46.4)	
4	6 (3.7)	4 (3.8)	2 (3.6)	
NE	35 (21.7)	21 (20.0)	14 (25.0)	
N, n (%)				
Negative	75 (46.6)	54 (52.4)	21 (37.5)	1.0
Positive	18 (11.2)	13 (12.4)	5 (9.9)	
NE	68 (42.2)	38 (36.2)	30 (53.6)	
M, n (%)				
0	51 (31.7)	34 (32.4)	17 (30.4)	0.375
1	45 (28.0)	34 (32.4)	11 (19.6)	
NE	65 (40.4)	37 (35.2%)	28 (17.4)	
ECOG, n (%)				
0	110 (68.3)	75 (71.4)	35 (62.5%)	0.547
≥1	14 (8.7)	8 (7.7)	6 (10.7)	
NE	37 (23.0)	22 (13.7)	15 (26.8)	
MSKCC, n (%)				
Favorable	16 (9.9)	8 (7.6)	8 (14.3)	0.584
Intermediate	50 (31.1)	30 (28.6)	20 (35.7)	
Unfavorable	5 (3.1)	2 (1.9)	3 (5.4)	
NE	90 (55.9)	65 (61.9)	25 (44.6)	
Metastatic sites, n (%)				
1	57 (35.4)	44 (41.9)	13 (23.2)	0.225
>1	62 (38.5)	41 (39.0)	21 (37.5)	
NE	42 (26.1)	20 (19.0)	22 (39.3)	
Cytokine, n (%)				
Yes	62 (38.5)	44 (41.9)	18 (32.1)	0.236
No	98 (60.9)	60 (57.1)	38 (67.9)	
NE	1 (0.6)	1 (1.0)	0 (0)	
PD ≤ 6 months, n (%)				
Yes	38 (23.6)	23 (21.9)	15 (26.8)	0.063
No	58 (36.0)	46 (43.8)	12 (21.4)	
NE	65 (40.4)	36 (34.3)	29 (51.8)	

NE: not evaluable, PD ≤ 6 months: progressive disease within 6 months of first-line therapy.

### Characteristics for second-line patients

One hundred and five patients (65%) received a second-line therapy, whereas 56 patients (35%) did not. Comparison of clinical and histopathological pretreatment baseline parameters between patients who did and did not receive second-line therapies failed to demonstrate a statistically significant difference (Table 1). However, analysis of histological grading showed that more patients with pretreatment grade 1 tumor received a second-line therapy than those with grade 2/3 tumors (P = 0.03; Table 1). Furthermore, patients without early progression tended to receive a second-line therapy more frequently than those who had an early progression (P = 0.063; Table 1).

### Second-line therapy is associated with a better OS

**Figure 1.**
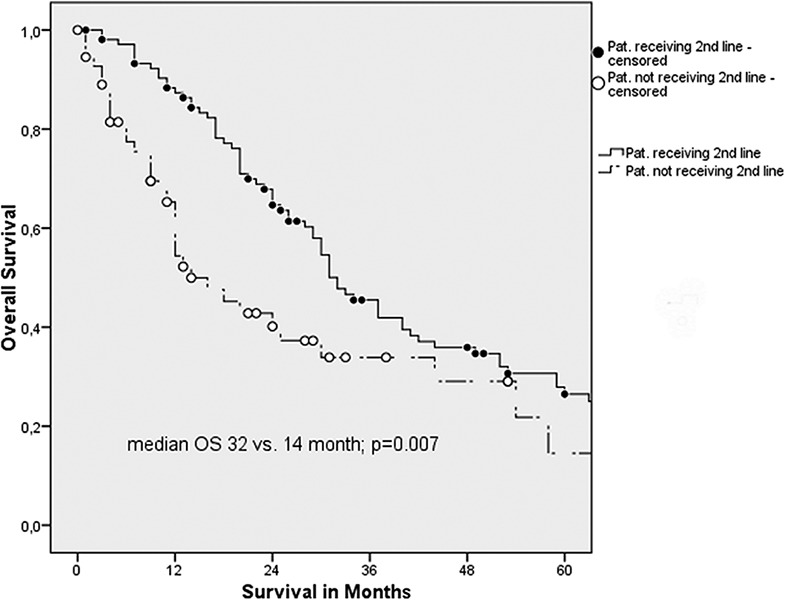
Kaplan–Meier analysis displaying improved median OS for patients receiving second-line therapy (32 versus 14 months; P = 0.007).

The median OS for all patients was 30 months (95% confidence interval [CI], 25.3–34.7). Kaplan–Meier analysis disclosed a median OS of 32 months (95% CI, 27.0–37.0) for patients receiving second-line therapy compared to patients who did not (median OS, 14 months; 95% CI, 8.4–19.6 months; log-rank: P = 0.007; Figure 1). In univariate analysis, application of second-line therapy was associated with better OS (HR, 1.75; 95% CI, 1.16–2.65; P = 0.008; Table 2). Additionally, the pretreatment grade 1 tumor was associated with improved median OS compared to pretreatment grade 2/3 tumors (130 months [95% CI, 25.7–234.3] versus 29 months [95% CI, 24.2–33.8]; log-rank P = 0.009; HR, 3.85; Table 2). Other factors such as ECOG performance status, MSKCC risk score, the presence of synchronous metastases, prior cytokine use, and early progression were also shown to be prognostic factors (Table 2). No independent risk parameter could be identified in multivariate analysis.

**Table 2.** Univariate analysis of patient characteristics related to OS

	OS, months, median (95% CI)	HR (95% CI)	*P*-value
M			
0 (indicator)	40 (28.4–51.6)	1.69 (1.04–2.73)	0.034
1	25 (17.6–32.4)		
ECOG			
0 (indicator)	30 (24.2–35.8)	3.82 (2.05–7.13)	<0.001
≥1	9 (6.3–11.8)		
MSKCC			
Favorable	37 (28.4–45.6)		
Intermediate	16 (9.7–22.3)	1.84 (0.89–3.82)	0.102 (favorable versus intermediate)
Unfavorable	4 (1.9–6.1)	2.26 (1.23–4.15)	0.009 (favorable versus un favorable)
Unknown	32 (23.7–40.3)	0.97 (0.77–1.23)	0.817 (favorable versus unknown)
PD ≤ 6 months			
No (indicator)	41 (24.4–57.6)	5.04 (3.06–8.31)	<0.001
Yes	11 (6.4–15.6)		
Metastatic sites			
1 (indicator)	30 (22.4–37.6)	1.48 (0.98–2.24)	0.062
>1	30 (22.0–38.0)		
Cytokine			
Yes (indicator)	49 (35.5–62.5)	2.73 (1.78–4.18)	<0.001
No	20 (14.5–25.5)		
TKI second line			
Yes (indicator)	32 (27.0–37.0)	1.75 (1.16–2.65)	0.008
No	14 (8.4–19.6)		
Grading			
G1	130 (25.7–234.3)	3.85 (1.40–10.63)	0.009
G2/3	29 (24.2–33.8)		

PD ≤ 6 months: progressive disease within 6 months of first-line therapy.

### Prognostic value of patient characteristics on second-line treatment administration

We tested all clinical parameters displayed in Table 1 according to the impact of second-line treatment administration by binary logistic regression. Early progression, defined as progression within 6 months of the start of first-line therapy, was significantly associated with an increased risk of not being able to receive a second-line therapy (HR, 2.50; 95% CI, 1.01–6.21; P = 0.048). However, none of the parameters were identified as independent risk parameter in the multivariate analysis.

## Discussion

The aim of this study was to identify prognostic baseline parameters that might help to identify whether mRCC patients would receive a second-line therapy. Currently, in Europe, mRCC patients have access to an armamentarium of 11 active agents; however, there is an increasing body of evidence that only a minority of patients enter a second-line therapy (12). The reasons for the high dropout rate before patients enter second-line therapy are largely unknown. However, death related to rapid tumor progression during first-line therapy does not explain this phenomenon sufficiently (12). Knowledge about baseline parameters of patients who will be able to enter a second-line therapy is an important issue, in particular, because immunotherapeutic agents could soon be introduced in the first-line setting. This will confront treating physicians in the near future with an increased challenge to choose the right agent for the right patient.

Our results show a significant difference in baseline characteristics between patients who received second-line therapy and patients who did not. Grade 1 tumors were significantly more frequent in patients who received second-line therapy. In fact, grade 1 tumors were found only in patients who received the second-line therapy (8.6% versus 0.0%; P = 0.03). Correspondingly, the OS of mRCC patients with G1 tumors was significantly longer than those with G2/3 tumors (median OS in months, 130 [95% CI, 25.7–234.3] versus 29 [95% CI, 24.2–33.8]; log-rank P = 0.009; HR, 3.85). Nonetheless, this parameter was not identified as an independent risk predictor for second-line receiver. Therefore, we hypothesize that higher grading might be associated with a likelihood for a patient not receiving a second-line therapy and vice versa. Additionally, early progression was an impediment for patients to receive a second-line therapy. In univariate analysis, early progression was the only parameter significantly associated with diminished OS and a higher risk of not being able to receive a second-line therapy (HR, 2.50; 95% CI, 1.01–6.21; P = 0.048). However, this parameter was not identified as an independent risk predictor for second-line receiver. The retrospective nature of a single-center analysis is naturally limited and most likely carries a selection bias. Limited sample number, missing data, and retrospective data acquisition represent the major limitations of this study, potentially over- or underpowering the current identified trends. However, we suggest that our analysis does at least have a character of hypothesis generation.

Previous studies have shown that grading is a valuable prognostic tool in mRCC and predicts OS (12–14). The study of Sacré et al. (15) identified grading to be a prognostic factor at the start of second-line therapy. To our knowledge, there is no study so far that has identified pretreatment tumor grade as the predictor of eligibility to receive second-line therapy. Although preliminary with limited samples from a single center, these findings warrant further exploration to elucidate the prognostic significance of grading toward clinical decision making. Patients with an aggressive tumor biology might benefit from a specific therapeutic approach that has yet to be defined.

Furthermore, early progression could be identified as a parameter of prognostic value in the prediction of patients who will be able to receive second-line therapy. This notion is supported by previous findings. For example, the depth of remission during first-line therapy proved to be an independent prognostic factor. Early tumor shrinkage and the depth of remission were significantly associated with an improved OS (16). However, early progression itself is technically not a baseline parameter, which will generate problems because clinicians cannot base therapeutic decision making on this parameter up front of first-line therapy. Other prognostic markers and clinical scoring systems reliably predict OS ahead of first-line therapy. The original MSKCC score, the International Kidney Cancer Working Group, and the IMDC score are tools that are utilized in the clinic (17). By reliably predicting OS, these scores might guide us in therapeutic decision making. However, these parameters are not validated to predict the likelihood to receive a second-line therapy.

Keeping these in mind, reviews about sequential therapies and decision making have been extensively published (18–20). There are international and national guidelines, as well as reliable phase 3 studies on therapy of RCC. Moreover, various studies focused on predictive and prognostic markers of RCC. For example, a retrospective study of Al-Marrawi et al. (21) showed that response to second-line VEGF therapy does not depend on response to first-line therapy. Another study concluded that an early reduction in neutrophil-to-lymphocyte ratio indicates a survival benefit (21). Various studies also attempted to predict response to VEGF-targeted therapies by focusing on circulating proteins, tissue-based molecules, germ line polymorphisms, and genomic biomarkers. However, no definitive biomarker has yet been integrated into the clinical decision making in therapy naive mRCC patients (22, 23). These studies may give advice on clinical decision making but cannot predict the likelihood of a patient requiring second-line therapy. In contrast, our retrospective study is the first that identified pretreatment parameters predicting the eligibility to receive a second-line therapy.

In conclusion, our results support the hypothesis that patients with a high-grade tumor inherit poor prognosis, which might be associated with a likelihood not to receive a second-line therapy. Importantly, this study identifies early progression to be a parameter of prognostic value identifying patients who will not receive a second-line therapy. However, further research is necessary to elucidate the role of grading and early progression in RCC to identify patients with a high likelihood of receiving a second-line therapy.

## Conflicts of interest

V. Grünwald has received honoraria from BMS, Novartis, and Pfizer. He has received fees as an advisor and speaker from BMS, Novartis, Pfizer, and Bayer. P. Ivanyi received advisory and speaker fees from Novartis, GSK, Bayer, BMS, and Pfizer. All other authors have no conflicts of interest to declare.
